# Regressed Non-metastatic Germ Cell Tumour: A Case Report and Literature Review

**DOI:** 10.7759/cureus.47851

**Published:** 2023-10-28

**Authors:** Ahmed Abdalla, Omar Abdalla, Mostafa Shendy, Margaret Lyttle

**Affiliations:** 1 Urology, Mid Cheshire Hospitals NHS Foundation Trust, Crewe, GBR; 2 General Surgery, Mid Cheshire Hospitals NHS Foundation Trust, Crewe, GBR

**Keywords:** colored flow doppler ultrasound, urological cancer, testicular cancer metastasis, embryonal cell carcinoma, spontaneous regression of cancer, testicular germ cell tumors

## Abstract

Testicular germ cell tumour regression is a rare phenomenon, where the primary testicular tumour spontaneously regresses, typically with metastatic disease at presentation. We present a case of a regressed germ cell tumour (GCT) in a 44-year-old post-pubertal male. Initially treated for suspected infection, the patient's testicular swelling prompted further investigation, leading to a radical orchidectomy that revealed the unusual histomorphologic findings of an entirely necrotic, non-seminomatous GCT consistent with a pure embryonal carcinoma.

## Introduction

The World Health Organization classifies testicular tumours into germ cell tumours (GCT) derived from germ cell neoplasia in situ (GCNIS), GCT unrelated to GCNIS, sex cord-stromal tumours, and tumours of the testicular adnexa [1].

A rare type of GCT derived from GCNIS is the regressed or ‘burned out’ GCT, which is a primary testicular GCT that spontaneously regresses even if, typically, there is metastatic disease at presentation [2].

Regression can occur in all types of testicular GCT, although it is most frequently seen in seminomas where the regressed tumour is replaced by a discrete fibrous nodule or scar [3].

We present a non-metastatic regressed non-seminomatous embryonal carcinoma GCT in a post-pubertal male.

## Case presentation

A 44-year-old, fit and well, man presented with a four-day history of a firm tender left testicular swelling. This was initially treated as orchitis with antibiotics, and an ultrasound scan (USS) of the testes was arranged as a follow-up. Testing for sexually transmitted infections was not conducted in accordance with the patient's preferences.

**Figure 1:**
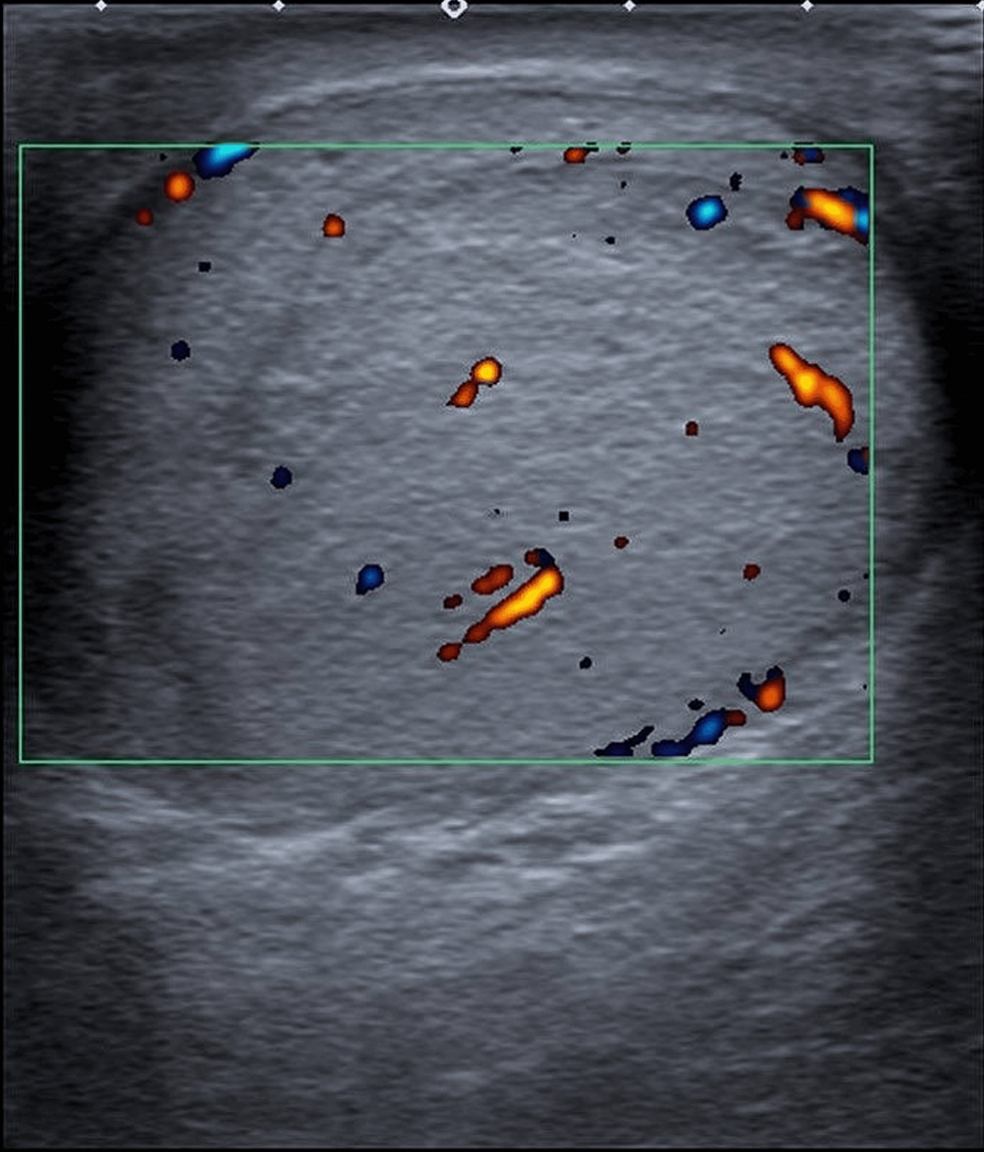
Doppler USS of the left testis showing enlargement and heterogenous echotexture.

Two weeks later, a Doppler ultrasound (USS, Figure 1) showed that his testis appeared enlarged and heterogenous in echotexture, with appearances raising suspicion of orchitis. However, an underlying focal intratesticular lesion could not be completely excluded.

**Figure 2:**
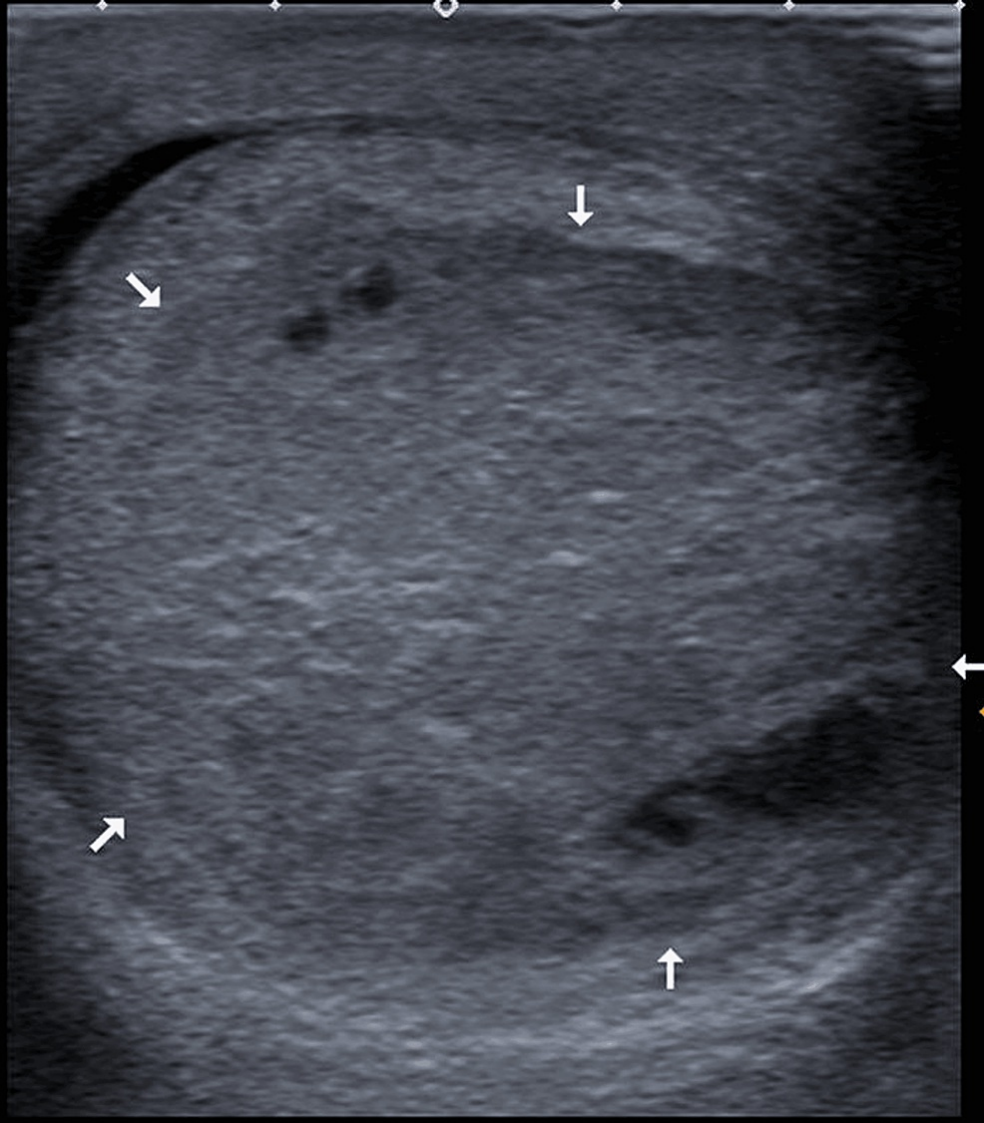
USS of left testis showing an increase in the size of the lesion.

The clinical presentation and imaging were reviewed at the uro-radiology multidisciplinary team (MDT) meeting, which suggested a repeat USS (Figure 2). This, in turn, showed an increasing size of a lesion in the now-painless left testis. There were appearances of cystic areas, concerning the possibility of malignancy. 

In light of the new imaging, the case was re-discussed at the uro-radiology MDT meeting, which advised offering radical orchidectomy for histology and staging CT chest, abdomen, and pelvis.

Additional preoperative investigations included serum tumour markers, which were within the normal range; LDH (lactate dehydrogenase) 493 iu/L (normal range 250-500 iu/L), AFP (alpha-fetoprotein) 10 ku/L (normal range 0-10 ku/L), and B-HCG (beta human chorionic gonadotropin) <1 iu/L (normal range 0-5 iu/L).

A staging contrast-enhanced CT of the chest, abdomen, and pelvis was conducted to assess the presence of metastatic disease. This showed a 2 mm round right apical lung nodule of indeterminate significance. Otherwise, there were no other significant findings. 

The patient underwent an uneventful left radical orchidectomy without the insertion of a prosthesis, followed by an uncomplicated postoperative recovery. 

Postoperatively, the case was discussed at the uro-oncology MDT meeting to review the histology. The MDT meeting also advised interval chest CT in three months for lung nodule follow-up and referral for regional testis MDT meeting discussion. 

Histopathology revealed that the entire testis was replaced by a solid well-circumscribed nodule with a tan-coloured cut surface and occasional haemorrhagic areas, where there was no viable tumour. On microscopy, the nodule described was processed in its entirety where it was composed of necrotic tissue with foci of haemorrhage, with a peripheral rim of hyalinised fibrosis mixed with macrophages and chronic inflammatory cells. The surrounding seminiferous tubules show atrophy, and there is focal GCNIS. The epididymis shows patchy foreign body type inflammation within the stroma. Immunohistochemical (IHC) stains showed that most of the necrotic cells within the nodule expressed Oct-3/4 and CD30, although staining for CD117, AFP, and HCG stains was negative, supporting a non-seminomatous embryonal carcinoma. The tumour was staged as pT1 with GCNIS present within the limits of the absence of a viable tumour.

In summary, the appearances were in keeping with a regressed germ cell tumour, with evidence supporting non-seminomatous embryonal carcinoma. The presence of other non-seminomatous and seminomatous components cannot be eliminated. There is no evidence of spread outside of the testis or lymph-vascular invasion and the spermatic cord and epididymis appear clear.

Regional testis MDT meeting recommended offering surveillance with interval examination and cross-sectional imaging.

Moreover, interval chest CT at three months and nine months showed regressing appearances of the lung nodule, which was deemed to not be for further surveillance after a review by the respiratory physicians.

## Discussion

A literature review shows that pure seminoma is considered the most common subtype in regressed GCTs. This is thought to be primarily because of its prevalence among testicular GCTs, rather than a specific inclination for regression in seminoma [3-5]. This is illustrated by the fact that the H&E histomorphology and IHC stains in our case showed a necrotic tumour, consistent with a non-seminomatous GCT, specifically embryonal carcinoma. Identifying the specific subtype of a regressed GCT from remaining features in the testis is challenging; however, ghost tubules and intratubular coarse calcifications may provide hints about the original tumour type [2-4].

It has been stressed that even small scars should be thoroughly examined for GCT regression, as they can be easily overlooked. Patients with clinical evidence of GCT but negative sampling should consider the possibility of a regressed tumour in the opposite testis or an extragonadal primary [5].

Additionally, patients with regressed GCT may also experience reduced spermatogenesis and testicular microlithiasis. Distinguishing between primary and secondary neoplasms is crucial when dealing with extragonadal germ cell tumours, particularly retroperitoneal ones, emphasizing the importance of comprehensive testicular assessment. Metastatic findings from ‘burned-out’ tissue have a prognosis similar to primary testicular germ cell tumours [5].

In 2006, Balzer et al. found that the presence of intratubular germ cell neoplasia unspecified (IGCNU), now called focal germ cell neoplasia in situ, is the most specific indicator of regression in germ cell tumours within scarred testes, even without known metastatic GCT. Another relatively specific sign of GCT regression is the presence of coarse, irregular intratubular calcifications related to regressed intratubular embryonal carcinoma. Care must be taken not to confuse these with smaller, round calcifications found in atrophic tubules [2].

In cases without IGCNU/GCNIS or coarse calcifications but displaying signs of regression, additional features such as numerous small vessels within the scarred area, clusters of Leydig cells, atrophic seminiferous tubules, and a lymphoplasmacytic infiltrate support the diagnosis of GCT regression [2]. In this case, the left testis showed atrophy and GCNIS.

Other tumours where spontaneous regression (SR) can be observed (although less commonly than in GCT) are renal cell carcinoma and melanoma. It was suggested that SR factors are linked to immune responses and blood supply disruption, with activated immune response likely being a primary trigger and as the testes are typically an immunoprivileged site, immunological anti-tumour responses may be one reason why GCTs are more prone to SR [6,7].

Ultrasound examination typically reveals residual tumour scars with varying sonographic appearances [5].

Orchidectomy is often required for histological confirmation and removal of potentially viable tumour cells that may not be fully treated by systemic chemotherapy, reducing the risk of tumour relapse. Spontaneous tumour regression in burnt-out testicular tumours may be because of factors such as a tumour outgrowing its blood supply, immunological reactions, and elimination [2,3,5].

## Conclusions

In conclusion, germ cell tumour regression, defined as the partial or complete disappearance of tumour with or without evidence of metastatic spread, is an important phenomenon that urologists and pathologists along with other members of the multidisciplinary team should be aware of.

Recognizing this phenomenon clinically, radiologically, and pathologically will enhance the management of these cases and ultimately benefit patient outcomes.
